# Comparison of CURB-65, PSI, and qSOFA for predicting pneumonia mortality in patients with idiopathic pulmonary fibrosis

**DOI:** 10.1038/s41598-021-83381-z

**Published:** 2021-02-16

**Authors:** Ryo Yamazaki, Osamu Nishiyama, Kazuya Yoshikawa, Sho Saeki, Hiroyuki Sano, Takashi Iwanaga, Yuji Tohda

**Affiliations:** Department of Respiratory Medicine and Allergology, Faculty of Medicine, Kindai University, Osakasayama, Osaka 589-8511 Japan

**Keywords:** Outcomes research, Risk factors

## Abstract

Some patients with idiopathic pulmonary fibrosis (IPF) require hospitalization due to pneumonia. Although predictive scoring tools have been developed and validated for community-acquired pneumonia (CAP), their usefulness in IPF is unknown. The Confusion, Urea, Respiratory Rate, Blood Pressure and Age (CURB-65) score and the Pneumonia Severity Index (PSI) are validated for CAP. The quick Sequential Organ Failure Assessment (qSOFA) is also reported to be useful. The aim of this study was to investigate the ability of these tools to predict pneumonia mortality among hospitalized patients with IPF. A total of 79 patients with IPF and pneumonia were hospitalized for the first time between January 2008 and December 2017. The hospital mortality rate was 15.1%. A univariate logistic regression analysis revealed that the CURB-65 (odds ratio 4.04, 95% confidence interval 1.60–10.2, *p* = 0.003), PSI (4.00, 1.48–10.7, 0.006), and qSOFA (5.00, 1.44–1.72, 0.01) scores were significantly associated with hospital mortality. There was no statistically significant difference between the three receiver operating characteristic curves (0.712, 0.736, and 0.692, respectively). The CURB-65, PSI, and qSOFA are useful tools for predicting pneumonia mortality among hospitalized patients with IPF. Because of its simplicity, the qSOFA may be most suitable for early assessment.

## Introduction

Idiopathic pulmonary fibrosis (IPF) is the most frequent cause of idiopathic interstitial pneumonia (IIP). IPF is a chronic and progressive lung disease with a poor prognosis, although the clinical course is highly variable^1,2^. Many patients with IPF require respiratory-related hospitalization (RH)^3^. RH is an important clinical event in IPF, because it is associated significantly with in-hospital and post-discharge mortality^4,5^.

Pneumonia is one of the major reasons for RH in patients with IPF. Cottin et al. reported using a discharge summary at a French hospital to determine that pulmonary infection occurred in 43.7% of patients with IPF who required RH^6^. Moreover, pulmonary infection of patients with IPF is reported to be associated with a high mortality rate, ranging from 18 to 30%^6,7^. Hence, pneumonia is important in the clinical course of IPF. Predicting pneumonia mortality in patients with IPF is crucial.

Several tools have been developed for the assessment of pneumonia severity, such as the Confusion, Urea, Respiratory Rate, Blood Pressure and Age (CURB-65) score and the Pneumonia Severity Index (PSI)^8,9^. For diagnosing sepsis, the Sepsis-3 Task Force proposed a scoring system that is simple and easy, i.e., the quick Sequential Organ Failure Assessment (qSOFA)^10^. A previous study demonstrated that the qSOFA can be used as a prognostic tool for patients with community-acquired pneumonia (CAP) who require hospitalization^11^. Based on these findings, we examined whether these scoring tools could predict pneumonia mortality in patients with IPF as well.

## Results

During the study period, 79 patients (61 men and 18 women) with IPF were hospitalized for pneumonia for the first time. The baseline clinical characteristics of the patients and treatment for IPF prior to hospitalization are shown in Table 1. The mean age was 74.6 ± 5.7 years. The mean forced vital capacity (FVC) was 68.9 ± 23.8% predicted, and the mean diffusing capacity for carbon monoxide (DLco) was 58.2 ± 17.4% predicted. The clinical blood sampling data at the time of hospitalization are shown in Table 2. The mean C-reactive protein level was 14.9 ± 0.2 mg/dL, mean white blood cell count 11,769 ± 4386/µL, mean platelet counts 26.7 ± 9.9 × 10^4^/µL, and mean partial pressure of arterial oxygen/fraction of inspiratory oxygen (PaO_2_/FiO_2_) 269 ± 80. Twenty-four of the 79 patients (30.3%) had bilateral lung involvement. Causative pathogens were detected in 23 patients (29.1%). The most common pathogen was *Haemophilus influenzae* (10.1%), followed by *Branhamella catarrhalis* (3.7%), *Klebsiella pneumoniae* (3.7%), and *Pseudomonas aeruginosa* (3.7%). As for survival, the 30-day and total hospital mortality rates were 12.6% and 15.1%, respectively. The mean duration of hospitalization was 25.8 ± 40.7 days. Univariate logistic regression analysis revealed that the CURB-65 (odds ratio [OR] 4.09, 95% confidence interval [CI] 1.60–10.2, *p* = 0.003), PSI (OR 4.00, 95% CI 1.48–10.7 *p* = 0.006), and qSOFA (OR 5.00, 95% CI 1.44–1.72, *p* = 0.01) were significantly associated with pneumonia mortality in hospitalized patients with IPF (Table 3). Regarding other variables, the PaO_2_ /FiO_2_ ratio (OR 0.99, 95% CI 0.98–0.99, *p* = 0.01), SOFA (OR 1.83, 95% CI 1.22–2.75, *p* = 0.003), sepsis (SOFA score ≥ 2) (OR 2.54, 95% CI 1.29–4.97, *p* = 0.006), and APACHE II (OR 1.12, 95% CI 1.00–1.26, *p* = 0.03) were significantly associated with hospital mortality (Table 3). When the data were adjusted for age, gender, and comorbidities (the Charlson comorbidity index^12^), the CURB-65 (OR 4.60, 95% CI 1.61–13.1 *p* = 0.004), PSI (OR 5.15, 95% CI 1.48–17.8 *p* = 0.009), and qSOFA (OR 5.12, 95% CI 1.33–19.6, *p* = 0.01) were still significant. The relationship between each severity score and hospital mortality is shown in Table 4. A similar result was observed for each severity score, in that the risk of hospital mortality rose as each score worsened.

**Table 1:** Patient baseline characteristics and treatment for IPF.

Variables	Values (n = 79)
Age, years	74.6 ± 5.7
**Gender**	
Male/female	61/18
Body mass index ^a^, kg/m^2^	20.7 ± 3.8
**Pulmonary function tests**	
FVC ^b^, L	2.0 ± 0.5
FVC ^b^, % predicted	68.9 ± 23.8
DLco ^c^, mL/min/mmHg	8.2 ± 2.5
DLco ^c^, % predicted	58.2 ± 17.4
**Smoking status**	
Current/Former/Never	4/61/14
**Treatment of IPF at baseline**	
Pirfenidone	9
Nintedanib	1
Corticosteroid	19
Cyclosporine	8
None	51
**Long-term oxygen therapy**	
Yes/no	30/49
**Comorbidities**	
Coronary artery disease	16
Hypertension	39
Diabetes mellitus	25
Dyslipidemia	16
Atrial fibrillation/flutter	7
Charlson comorbidity index	1.7 ± 1.0

The values are expressed as mean ± standard deviation or actual number.

*DLco* diffusing capacity for carbon monoxide; *FVC* forced vital capacity; *IPF* idiopathic pulmonary fibrosis.

^a^n = 77; ^b^n = 52; ^c^n = 32.

**Table 2:** Patient clinical data at the first hospitalization.

Variables	Values (n = 79)
**Vital signs**	
Level of consciousness (GCS)	14.9 ± 0.2
Heart rate, /min	97 ± 18
sBlood pressure, mmHg	121 ± 19
dBlood pressure, mmHg	69 ± 13
Mean blood pressure, mmHg	86 ± 13
Respiratory rate, /min	24 ± 6
Temperature, °C	37.3 ± 1.0
**Laboratory data**	
CRP, mg/dL	11.8 ± 8.6
WBC, /µL	11,769 ± 4386
Platelet count, × 10^4^/µL	26.7 ± 9.9
BUN, mg/dL	20 ± 19
Cr, mg/dL	1.0 ± 1.4
PT-INR ^a^	1.15 ± 0.18
Fibrinogen^b^, mg/µL	551 ± 196
FDP^c^, μg/mL	9.6 ± 20.0
D-dimer^d^, µg/mL	3.76 ± 6.8
KL-6^e^, U/mL	945 ± 596
**Arterial blood gas test**	
pH	7.42 ± 0.05
PaO_2_/FiO_2_ ratio	269 ± 80
PaCO_2_, torr	39.4 ± 9.1
**Illness severity**	
CURB-65	1.4 ± 0.7
PSI	3.2 ± 0.7
qSOFA	0.6 ± 0.6
SOFA	1.9 ± 0.9
APACHE II score	8.8 ± 4.8
NIPPV, y/n	5/74
NHF, y/n	2/77
Mechanical ventilation, y/n	3/76

Values are expressed as mean ± standard deviation or actual number.

*APACHE II* Acute Physiology and Chronic Health Evaluation II; BUN, blood urea nitrogen; *Cr* creatinine; *CRP* C-reactive protein; *CURB-65* confusion, urea, respiratory rate, blood pressure and age score; *FDP* fibrinogen and fibrin degradation products; *GCS* Glasgow coma scale; *KL-6* Krebs von der Lungen-6; *NHF* nasal high flow; *NIPPV* noninvasive positive pressure ventilation; *PaCO*_*2*_ partial pressure of carbon dioxide; *PaO*_*2*_*/FiO*_*2*_ partial pressure of atrial oxygen / fraction of inspiratory oxygen; *PSI* Pneumonia Severity Index; *PT INR* prothrombin tome-international normalized ratio; *qSOFA* quick Sequential Organ Failure Assessment; *SOFA* sequential organ failure assessment; *WBC* white blood cell.

^a^n = 74; ^b^n = 48; ^c^n = 62; ^d^n = 54; ^e^n = 77.

**Table 3:** Results of the univariate logistic regression analysis of hospital mortality (n = 79).

Variable	Odds ratio	95% CI	*p* value
Age, per year	1.03	0.91–1.09	0.49
Female, sex	0.63	0.12–3.21	0.58
Body mass index^a^	0.85	0.70–1.03	0.10
FVC^b^	0.92	0.25–3.33	0.90
FVC, % predicted^b^	0.99	0.94–1.03	0.63
DLco^c^	0.77	0.34–1.72	0.53
DLco, % predicted^c^	0.93	0.79–1.08	0.36
CRP	0.96	0.89–1.04	0.38
WBC	1.00	1.00–1.00	0.58
Platelet counts	0.97	0.90–1.04	0.43
Fibrinogen^d^	0.99	0.99–1.00	0.20
FDP^e^	1.10	0.97–1.24	0.12
D-dimer^f^	1.29	0.98–1.69	0.06
KL-6^g^	1.00	0.99–1.00	0.68
PaO_2_/FiO_2_	0.99	0.98–0.99	0.01
PaCO_2_	1.06	0.99–1.12	0.05
CURB-65	4.04	1.60–10.2	0.003
PSI	4.00	1.48–10.7	0.006
qSOFA	5.00	1.44–1.72	0.01
SOFA	1.83	1.22–2.75	0.003
Sepsis (SOFA score ≥ 2)	2.54	1.29–4.97	0.006
APACHE II score	1.12	1.00–1.26	0.03

*APACHE II* Acute Physiology and Chronic Health Evaluation II; *CURB-65* confusion, urea, respiratory rate, blood pressure and age; *CRP* C reactive protein; *DLco* diffusing capacity for carbon monoxide; *FDP* fibrinogen and fibrin degradation products; *FVC* forced vital capacity; *KL-6* Krebs von der Lungen-6; *PaCO*_*2*_ partial pressure of carbon dioxide; *PaO*_*2*_*/FiO*_*2*_ partial pressure of arterial oxygen / fraction of inspiratory oxygen; *PSI* Pneumonia Severity Index; *qSOFA* quick Sequential Organ Failure Assessment; *SOFA* sequential organ failure assessment; *WBC* white blood cell.

^a^n = 77; ^b^n = 52; ^c^n = 32; ^d^n = 48; ^e^n = 62; ^f^n = 54; ^g^n = 77.

**Table 4:** Relationships between the three severity scores and hospital mortality (n = 79).

Severity score	Class	No. of patients	Hospital mortality
CURB-65	0	2	0%
	1	44	9.0%
	2	28	14.2%
	3	3	66.6%
	4	2	100%
PSI	I	0	0%
	II	13	0%
	III	38	10.5%
	IV	25	24.0%
	V	3	66.6%
qSOFA	0	30	6.0%
	1	45	22.5%
	2	3	66.6%
	3	1	100%

*CURB-65* confusion, urea, respiratory rate; blood pressure and age; *PSI* Pneumonia Severity Index; *qSOFA* quick Sequential Organ Failure Assessment.

**Figure 1:**
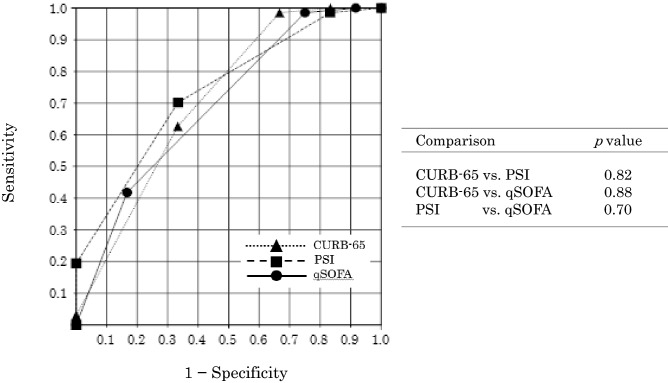
Comparison of the receiver operating characteristic (ROC) curves of the CURB-65, PSI, and qSOFA scoring systems for predicting pneumonia mortality in hospitalized patients with IPF. *CURB-65* Confusion, Urea, Respiratory Rate, Blood Pressure and Age; *PSI* Pneumonia Severity Index; *qSOFA* quick Sequential Organ Failure Assessment.

The ROC curves for hospital mortality are shown in Fig. 1. The clinical utility of the CURB-65, PSI, and qSOFA to predict in-hospital mortality is shown in Table 5. The qSOFA had a sensitivity and specificity (98.5% and 75.0%, respectively) higher than or equal to those of the CURB-65 and PSI. The PSI had the best discriminatory value (AUC 0.736; 95% CI 0.660–0.811), followed by the CURB-65 (AUC, 0.712; 95% CI 0.620–0.801), and the qSOFA (AUC, 0.692; 95% CI 0.602–0.779). However, there were no significant differences among the three scoring systems.

**Table 5:** Clinical utility of the three pneumonia severity scores for predicting in-hospital mortality (n = 79).

Severity tools	AUC (95% CI)	Cut-off threshold	Score category	Sensitivity (%)	Specificity (%)	PPV (%)	NPV (%)
CURB-65	0.712 (0.620–0.801)	2.06	CURB-65 ≥ 3	98.5	66.6	89.1	80.0
PSI	0.736 (0.660–0.811)	3.96	PSI ≥ 4	70.1	33.3	92.1	28.5
qSOFA	0.692 (0.602–0.779)	1.36	qSOFA ≥ 2	98.5	75.0	88.0	75.0

The cut-off threshold was extracted from the ROC curves.

*AUC* area under the receiver operating characteristic curve; *CI* confidence interval; *CURB-65* confusion, urea, respiratory rate, blood pressure and age score; *IPF* idiopathic pulmonary fibrosis; *NPV* negative predictive value; *PPV* positive predictive value; *PSI* Pneumonia Severity Index; *qSOFA* quick Sequential Organ Failure Assessment; *ROC* receiver operating characteristic.

## Discussion

The CURB-65 and PSI were developed to predict prognosis in patients with CAP. Although the qSOFA was proposed as a simple bedside scoring tool for early identification of sepsis, it has also been reported that this prognostic tool could be used in patients with CAP who required hospitalization^11^. To the best of our knowledge, this is the first study to show that these tools predict the survival of patients with IPF with pneumonia as well. Given its comparable discriminatory power with 2 existing tools, the qSOFA seems to be the best tool for assessment in the clinical setting.

The hospital mortality rate for CAP requiring hospitalization is reported to range from 2 to 8%^13,14^. However, pulmonary infection of patients with IPF is associated with a high mortality rate, ranging from 18 to 30%^6,7^. Our study showed that the pneumonia mortality rate of hospitalized patients with IPF was 15.1%. Hence, it is important to recognize that pneumonia is more lethal in patients with IPF than in patients without IPF. Therefore, discriminating patients who would die of pneumonia is crucial for patients with IPF and pneumonia.

As for the qSOFA, there were no statistically significant differences in ROC curves when the qSOFA ROC curve was compared with those of the CURB-65 and the PSI. However, the sensitivity of the qSOFA was higher than that of the other tools. It was reported that the sensitivity of the qSOFA ≥ 2 for mortality in patients hospitalized with CAP was 39.1–50%^11,15^. In this study, only a small number of patients had a qSOFA score of ≥ 2 points (5.0%) with extremely high hospital mortality (66.6–100%). This result might be associated with the high sensitivity of the qSOFA.

When using these tools for patients with pneumonia and IPF it is also important to take the characteristics of the three tools into account. The PSI may overestimate cancer which is unrelated to the lung such as prostate cancer. It may also overestimate the severity in elderly patients because it is heavily weighted towards age. The CURB-65 also includes age as a scoring variable, but only categorizes age as either ≧ 65 or not. The qSOFA does not include any age variable, resulting in possible underestimation in elderly patients.

This study had several limitations. First, it was performed at a single center and had a relatively small sample size. Because the criteria determining hospitalization are different between regions and countries there is a need for larger multicenter studies. Second, it was a retrospective study. Third, only patients with pneumonia who required hospital admission were included. If we had included patients who could have been treated in an outpatient clinic, the results might have been different. Fourth, pulmonary hypertension was not evaluated although it is an important prognostic complication in patients with IPF^16^. Finally, it is possible that the study might have included patients with acute exacerbation (AE) of IPF. The 2016 International Working Group proposed both idiopathic and triggered AE. Triggered AE includes those after infection, drug toxicity, aspiration, or post-procedure/post-operative^17^. However, validation of triggered AE has been not performed in a multicenter study. In this study, we made major efforts to exclude patients with triggered and suspected triggered AE after careful discussion involving several specialists. Despite our efforts, some patients may have been included.

In conclusion, three scoring tools, the CURB-65, PSI, and qSOFA can predict mortality from pneumonia in hospitalized patients with IPF. Discriminatory power was comparative among the three tools. Hence, the qSOFA would be useful in the clinical setting based on its simplicity.

## Methods

### Patients

From January 2008 through December 2017, we retrospectively reviewed the medical data of all patients with IPF who required admission to the Kindai University Hospital for pneumonia. IPF was diagnosed based on a recent official statement^1^. Pneumonia was defined as: (1) fever, productive cough, or abnormal white blood cell count, and (2) newly developed consolidation and/or ground-glass opacities on a chest radiograph or chest high-resolution computed tomography (HRCT). The study protocol was approved by the ethics committee of the Kindai University Faculty of Medicine (No. 31-244). Informed consent was waived, because this study was based on a retrospective analysis of case records from our university hospital. All methods were performed in accordance with the relevant guidelines and regulations (Declaration of Helsinki).

### Pulmonary function tests

The most recent pulmonary function tests (PFT) performed within 1 year prior to the diagnosis of pneumonia were used to establish baseline pulmonary function. The PFT were performed using a CHESTAC-8800 (Chest, Tokyo, Japan) according to the standards proposed by the European Respiratory Society^18,19^.

### Data collection

We assessed the baseline clinical characteristics of the patients including age, gender, smoking status, long-term oxygen therapy, and treatment for IPF. Routine blood sampling and standard laboratory techniques were carried out at admission. The Charlson Comorbidity Index was calculated to assess the extent of comorbidities^12^.

### Tools for predicting pneumonia mortality

The qSOFA score was calculated according to the Sepsis-3 Task Force scoring system. This score includes systolic blood pressure ≤ 100 mmHg, respiratory rate ≥ 22 breaths/min, and altered mental status. A total qSOFA score of ≥ 2 points indicates possible organ dysfunction^10^. The CURB-65 is a predictive tool for CAP recommended by the British Thoracic Society (BTS)^20^. The criteria include confusion status, blood urea nitrogen > 20 mg/dL, respiratory rate ≥ 30, systolic blood pressure < 90 mmHg or diastolic blood pressure ≤ 60 mmHg, and age ≥ 65 years^8^. In this study, patients who had a CURB-65 score of ≥ 3 points were classified as being at a high risk of death according to the BTS guidelines^20^. The PSI proposed in 1997 is a useful tool for predicting mortality in patients with CAP^9^. The PSI includes demographics, comorbidities, a physical examination, and laboratory and radiological findings. A PSI class of I–III was reported to represent a low risk of death^8^. In our study, patients who had a PSI class of ≥ IV were defined as being at a high risk of death.

### Assessment of survival

We evaluated the 30-day mortality and the total hospital mortality of the patients. All deaths were confirmed by hospital chart review.

### Statistical analysis

Continuous variables were expressed as means ± standard deviation (SD) and categorical variables as frequencies. Univariate and multivariate logistic regression analyses were used to identify potential risk factors for hospital mortality. The area under the receiver operating characteristic (ROC) curve (AUC) with a 95% confidence interval (CI) was used to assess discriminatory value. Z tests as described by Hanley and McNeil were used to compare pairs of ROC curves^21^. A *p* value of < 0.05 was considered statistically significant. The analyses were performed with Statflex ver.6 (Artech, Co., Ltd., Osaka, Japan).

## Data Availability

All data are available if requested.
